# Effectiveness and safety of low-level laser therapy in diabetic peripheral neuropathy: a protocol for a systematic review and meta-analysis

**DOI:** 10.1186/s13643-021-01656-y

**Published:** 2021-04-02

**Authors:** Lin Lin, Jingjing Li, Jingshan Lin, Shiheng Tang, Yuxia Li

**Affiliations:** School of Nursing, Shanghai University of Traditional Chinese Medicine, 1200 Cailun Road, Shanghai, 201203 China

**Keywords:** Diabetic peripheral neuropathy, Low-level laser therapy, Nerve conduction velocity

## Abstract

**Background:**

Diabetic peripheral neuropathy (DPN) is one of the most common chronic complications of diabetes mellitus. The main symptoms of DPN include numbness or pain in both extremities and paresthesia (such as formication or burning sensations), which greatly affect patients’ quality of life. Pharmacological treatments for DPN are associated with both uncertain therapeutic effects and adverse effects, as well as with high costs. Some clinical studies have reported that low-level laser therapy (LLLT) relieves clinical symptoms and improves nerve function in patients with DPN. We intend to conduct a systematic review and meta-analysis to further evaluate the effectiveness and safety of LLLT for DPN.

**Methods:**

The following electronic databases will be searched to retrieve literature from their inception until December 2020: MEDLINE (PubMed), Embase, Cochrane Central Register of Controlled Trials, Web of Science (the Science and Social Science Citation Index), CNKI, VIP, WanFang, and SinoMed. Simultaneously, clinical registration tests and gray literature will also be retrieved. Randomized controlled trials (RCTs) comparing LLLT with either sham LLLT, no (specific) treatment, or active conventional medical treatments will be included. The primary outcomes will be nerve conduction velocity as well as clinical scores that assess neurological function and related symptoms. The risk of bias of each study and quality of evidence will be assessed using the updated Cochrane Risk of Bias 2.0 tool and GRADE approach, respectively. A meta-analysis will then be conducted using Review Manager software version 5.3.

**Discussion:**

This study will integrate RCTs and analyze data to provide a detailed summary of the evidence relating to the effects and safety of LLLT in patients with DPN. LLLT will be compared with sham LLLT, no (specific) treatment, or active conventional medical treatments, especially in terms of neurological function, quality of life, and adverse events. In conclusion, this systematic review will generate evidence regarding the use of LLLT to treat DPN, in terms of both its efficacy and safety.

**Systematic review registration:**

This protocol was registered with the International Prospective Register of Systematic Reviews on April 2020 (registration number: CRD42020170625).

## Background

Diabetic peripheral neuropathy (DPN) refers to the symptoms and/or signs related to peripheral nerve dysfunction in patients with diabetes mellitus (DM), after excluding other causes. The prevalence of DPN among DM patients is approximately 50%, and DPN is not only a common chronic complication of DM, but is also the commonest cause of neuropathy [1, 2]. DPN mainly manifests as symptoms such as numbness or pain in both extremities as well as paresthesia (such as formication or burning sensations), which greatly affect patients’ quality of life. In addition, DPN plays a key role in the development of diabetic foot complications [3]. Approximately 15–20% of diabetic foot ulcers require amputation [4, 5]. Furthermore, DPN is associated with a large human and economic burden on both patients and the healthcare system [6, 7].

The mechanisms of DPN are not completely clear, but mainly involve activation of the polyol pathway, advanced glycation end products (AGEs), dyslipidemia, oxidative stress, and a lack of neurotrophic factors [8, 9]. Currently, the treatment of DPN usually includes basic treatments, such as controlling blood glucose levels and providing diet and exercise guidance for patients, and etiological treatments, such as medicine to improve blood circulation, neurotrophic medicine, medicine to correct metabolic disorders, and anti-oxidative stress medicine [10]. In addition, symptomatic treatment is used to relieve neuropathic pain [11]. However, pharmacotherapy is associated with both uncertain therapeutic effects and adverse effects, as well as high costs [12, 13]. Non-pharmacological treatments, such as low-level laser therapy (LLLT), have also been proposed to treat DPN by relieving clinical symptoms and improving nerve function [14, 15]. Compared with conventional medicines, LLLT has the advantages of being non-invasive and having almost no adverse side effects. LLLT may produce biological stimulation of the nervous system [16], can improve the function of damaged neurological tissue [17], and reduce inflammation [18]. Some clinical studies have demonstrated that LLLT has a positive effect on nerve conduction velocity (NCV) and clinical scores [14, 15, 19, 20], but few of these studies had a large sample size. A recent systematic study from Anju M et al. [21] that has been conducted to investigate the effect of LLLT on painful diabetic peripheral neuropathy with standard of care treatment. However, this systematic study did not provide a quantitative analysis of the included studies. Besides, studies that used intervention other than LLLT were excluded in this systematic review. Rather than active treatment alone, LLLT is increasingly being combined with active treatment to relieve symptoms and increase NCV in DPN.

Therefore, the aim of this study is to conduct a systematic review and meta-analysis to evaluate the safety and effectiveness of LLLT compared with active conventional treatment, sham LLLT, or no treatment, or as an additional treatment compared with active treatment alone, in patients with DPN.

## Methods

### Protocol registration

This is a systematic review protocol for clinical trials. The aim of this systematic review is to investigate the safety and effectiveness of LLLT compared with active conventional treatment, sham LLLT, or no treatment, or as an additional treatment compared with active treatment alone, in patients with DPN. This protocol was registered with the International Prospective Register of Systematic Reviews on April 2020 (registration number: CRD42020170625; http://www.crd.york.ac.uk/prospero/).

### Criteria for included studies

To be included in this analysis, studies will need to meet the following criteria regarding types of studies, participants, interventions, controls, and outcomes (Table 1).

**Table 1:** The inclusion criteria

	Inclusion criteria
**Study design**	RCTs
**Participants**	Adults (older than 18 years) with a clinically confirmed diagnosis of DPN
**Interventions**	Low-level laser therapy
**Comparisons**	Sham LLLT, no treatment, active conventional medical treatment
**Outcomes**	At least one of the following outcomes: NCV, clinical scores that assess neurological function and related symptoms, quality of life scales, pain assessment tools

#### Types of studies

The randomized controlled trials (RCTs) that focus on LLLT in patients with DPN will be included. In contrast, reviews, case reports, animal experiments, meeting abstracts, and any publications without primary data or an explicit description of the methods will be excluded. Besides, we will only include the articles written in Chinese or English.

#### Participants

Adults (older than 18 years) with a clinically confirmed diagnosis of DPN will be included. The diagnostic criteria refer to the standards established by the Chinese Medical Association Diabetes Branch [22] or American Diabetes Association [23, 24]. We will exclude any RCTs that investigated patients with other types of peripheral neuropathy. Besides, patients with severe heart disease, liver and kidney dysfunction, mental illness, or malignant tumors will be not included.

#### Interventions

Currently, lasers with an output power of less than 500 mW are referred to as low-level lasers in the field of medicine. Common medical LLLTs include (but are not limited to) He-Ne lasers, semiconductor lasers, and CO_2_ lasers. RCTs will be included if they compare LLLT with any of the following control interventions: sham LLLT, no (specific) treatment except conventional diabetes treatments, or active conventional medical treatment. RCTs will also be included if they evaluate LLLT as an addition to another active treatment.

A. LLLT vs. sham LLLT
B. LLLT vs. no treatment
C. LLLT vs. active conventional medical treatment
D. LLLT plus conventional medical treatment X vs. conventional medical treatment X alone

#### Outcomes

The major outcomes for this review include NCV and clinical scores that assess neurological function and related symptoms.

NCV consists of motor NCV and sural sensory NCV. Clinical scores that assess neurological function and related symptoms include (but are not limited to) the Michigan Neuropathy Screening Instrument, Toronto Clinical Scoring System, Total Symptom Score, Neuropathy Deficit Score, and Neuropathy Symptom Score.

Outcomes will be recorded in two time periods: (1) short-term follow-up (less than or equal to 3 months and closest to 8 weeks after randomization) and (2) if available, long-term follow-up (more than 3 months and closest to 6 months after randomization).

Minor outcomes at both the short- and long-term follow-up will consist of quality of life such as the Short Form36 (SF-36), EuroQol five dimensions questionnaire (EQ-5D), and NeuroQol; pain assessment tools such as visual analog scale (VAS), neuropathic pain scale (NPS), Neuropathic Pain Questionnaire (NPQ), Short-form McGill Pain Questionnaire (SF-MPQ), and Brief Pain Inventory for Diabetic Peripheral Neuropathy (BPI-DPN); and adverse events associated with LLLT as a list of events.

### Search strategy

#### Electronic searches

A systematic literature search will be conducted in the following electronic bibliographic databases: MEDLINE (PubMed), Embase, Cochrane Central Register of Controlled Trials, Web of Science (Science and Social Science Citation Index), Chinese National Knowledge Infrastructure (CNKI), VIP China Science and Technology Journal Database (VIP), WanFang Database, and SinoMed, from their inception to December 2020. Two sets of keywords were chosen to identify the pertinent papers. The first set assessed the target population. The second set specified the type of intervention. A wildcard symbol (*) was used for generalizing keywords typically characterized by varying suffixes. The search was performed by inserting logical conjunctions (AND/OR) between the sets. Search areas included the “Mesh,” “title,” and “abstract” fields (Table 2). The articles need to be written in English or Chinese.

**Table 2:** Search strategy for the MEDLINE (PubMed) database

**Diseases terms**	
1	"Diabetic Neuropathies"[Mesh]
2	"diabetic peripheral neuropath*"[Title/Abstract] OR "diabetic neuropath*"[Title/Abstract] OR "DPN"[Title/Abstract]
3	1 OR 2
**Intervention terms**	
4	"low-level light therapy"[MeSH Terms]
5	"Laser Irradiation, Low-Power"[Title/Abstract] OR "Irradiation, Low-Power Laser"[Title/Abstract] OR "Laser Irradiation, Low Power"[Title/Abstract] OR "Light Therap*, Low-Level"[Title/Abstract] OR "Low Level Light Therap*"[Title/Abstract] OR "Therap*, Low-Level Light"[Title/Abstract] OR "Photobiomodulation Therap*"[Title/Abstract] OR "Therap*, Photobiomodulation"[Title/Abstract] OR "Laser Therap*, Low-Level"[Title/Abstract] OR "Laser Therap*, Low Level"[Title/Abstract] OR "Low-Level Laser Therap*"[Title/Abstract] OR "Low Level Laser Therap*"[Title/Abstract] OR "Low-Power Laser Therap*"[Title/Abstract] OR "Low Power Laser Therap*"[Title/Abstract] OR "Laser Therap*, Low-Power Laser Therap*, Low Power"[Title/Abstract] OR "Low-Power Laser Therap*"[Title/Abstract] OR "Low-Power Laser Irradiation"[Title/Abstract] OR "Low Power Laser Irradiation"[Title/Abstract] OR "Laser Biostimulation"[Title/Abstract] OR "Biostimulation, Laser"[Title/Abstract] OR "Laser Phototherapy"[Title/Abstract] OR "Phototherapy, Laser"[Title/Abstract] OR "low intensity laser therap*"[Title/Abstract] OR "low energy laser therap*"[Title/Abstract] OR "LLLT"[Title/Abstract] OR "LILT"[Title/Abstract] OR "LELT"[Title/Abstract]
6	4 OR 5
Combination of terms	
7	3 AND 6
8	3 AND 6 Filters: Randomized Controlled Trial; Humans

#### Searching other resources

To overcome any deficiencies of the electronic databases, the following websites will also be searched for other clinical trial registries and gray literature about LLLT for DPN: Chinese Clinical Trial Registry (http://www.chictr.org.cn/), World Health Organization International Trials Registry Platform (www.who.int/trialsearch/Default.aspx), China Dissertations Database, GreyNet International (http://www.greynet.org), Grey Literature Report (http://www.Greylit.org/), Google Scholar (http://scholar.google.com/), Baidu Scholar (https://xueshu.baidu.com).

### Study selection

**Fig. 1:**
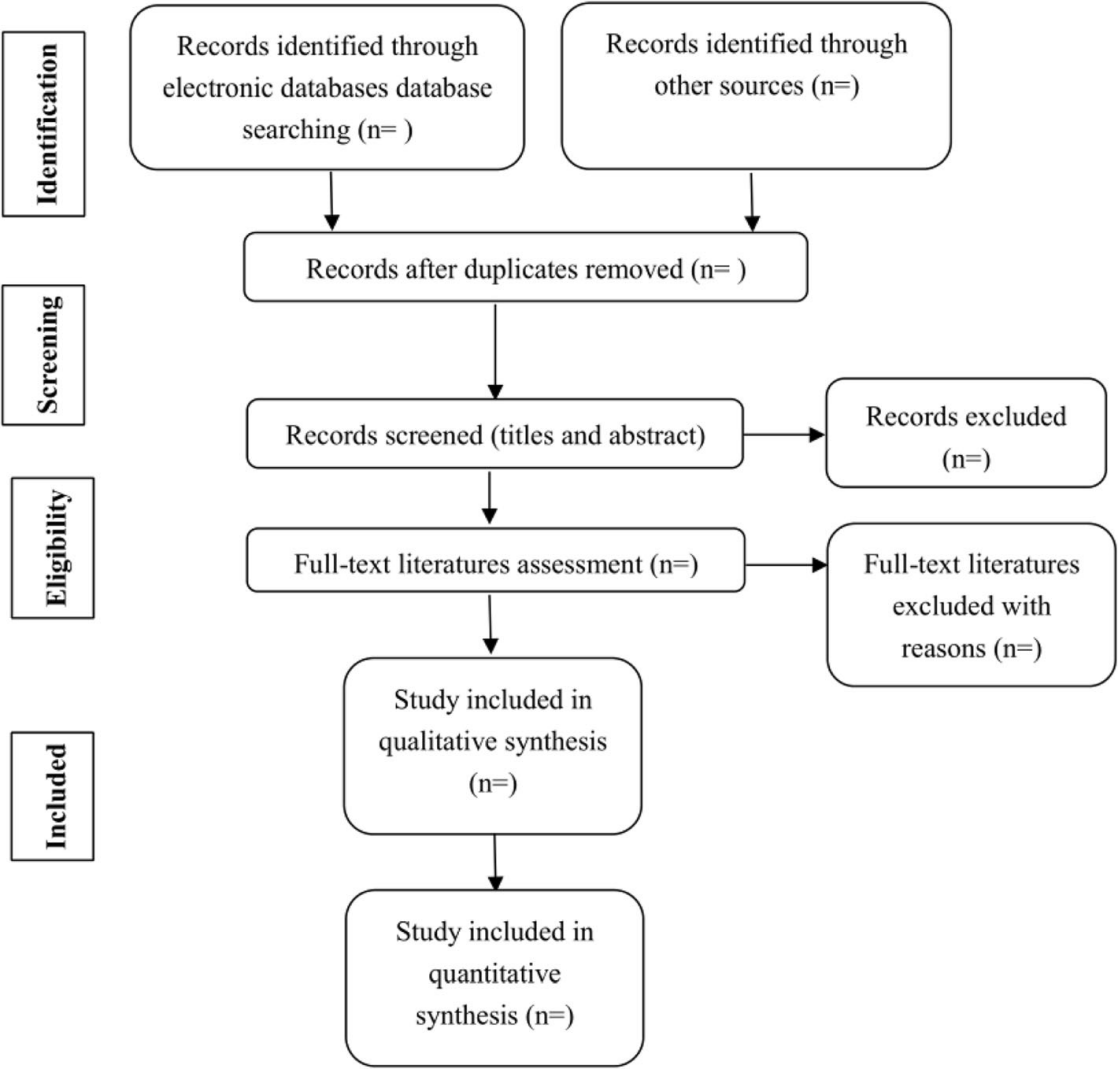
Flow chart of the search process

All data retrieved through the performed search will be imported into Endnote X7, and duplicate data from the different databases will be removed. Two reviewers will independently screen the title and abstract of each study and make a decision to include it or not, according to pre-specified criteria. Next, the full text of the initially included literature will be retrieved. Two reviewers will carefully read the full text and make the final selections. Any discrepancies between the two reviewers will be resolved through consensus and by third-party adjudication, as needed. The study selection procedure is shown in Fig. 1.

### Data extraction

The following data will be extracted from each included study: first author name, publication year, sample sizes, design, age, sex, BMI, diabetes duration, fasting blood glucose (FBG), postprandial blood glucose (2hPBG), glycosylated hemoglobin (HbA1c), DPN duration, intervention descriptions, comparators, treatment duration, follow-up periods, outcomes, and adverse events (Tables 3 and 4). Two reviewers will independently extract the data, and any differences will be solved by discussion. We will contact the authors of any studies that do not report the aforementioned data (via email) to obtain the original data. We will conduct sensitivity analyses if the missing data are still not obtained.

**Table 3:** General information of recruited studies

Reference	Sample	Design	Age	BMI	Gender	Diabetes duration/DPN duration	FBG	2hPBG	HbA1c
Authors, year	Number of participants	RCT, open label or blinded	Mean (SD), years	mean (SD), kg/m^2^	Male/female	Mean (SD), years	Mean (SD), mmol/L	Mean (SD), mmol/L	Mean (SD), %

**Table 4:** Characteristics of the intervention and outcomes

Reference	Intervention	Comparators	LLLT parameters			Therapeutic schedules	Treatment duration/follow-up periods	Outcomes	Adverse events
			Wavelength	Energy density/energy per point	Laser average Pulse				
Authors, year	LLLT alone or with active conventional medical treatment	Sham LLLT or no treatment or active conventional medical treatment	nm	J/cm^2^, J/point per session	mW	Treatment time/no. of total sessions/no. of sessions per week	Week	Measures of trial outcomes	Adverse events about LLLT

### Risk of bias assessment

Two reviewers will independently assess the risk of bias of each study using the updated Cochrane Risk of Bias 2.0 tool [25]. This tool consists of six domains (randomization process, intended interventions, missing outcome data, measurement of the outcome, selection of the reported result, and overall bias). Each separate domain will be rated as having a low risk of bias, some concerns, or a high risk of bias. Disagreements between the two reviewers will be resolved through consensus and by third-party adjudication, as needed [26].

### Strategy for data analysis

We will extract the main parameters of included articles according to the aim of this systematic review through Table 3, in which the article’s characteristics, methods, description of population, intervention descriptions, comparators, treatment duration, follow-up periods, outcomes, and adverse events will be included. After summarization of data, it will be determined if a meta-analysis is possible. If possible, Review Manager software version 5.3, provided by the Cochrane Collaboration, will be used to perform data synthesis and analysis. All experimental and control groups such as A, B, C, and D described in intervention will be evaluated as a single category.

#### Measures of treatment effect

We will select different evaluation methods according to the different types of data. Pooled dichotomous data will be presented as odds ratios or relative ratios with 95% confidence intervals. Pooled continuous data will be expressed as mean differences or standardized mean differences with 95% confidence intervals, depending on whether the measurement scale is consistent or not. Heterogeneity will be assessed by the chi-squared test and *I*^2^ statistic. If there is no heterogeneity (*P >* 0.1, *I*^*2*^ < 50%), the data will be synthesized using a fixed effect model. In contrast, a random-effects model will be used if (*P* < 0.1, *I*^*2*^ *>* 50%). Furthermore, we will use subgroup or sensitivity analyses to explore the potential reasons for the differences in heterogeneity. We will conduct a general descriptive analysis if a meta-analysis cannot be performed.

#### Assessment of heterogeneity

Statistical heterogeneity will be assessed by *I*^2^ statistic. When the *I*^2^ is greater than 50% (i.e., there is substantial heterogeneity), the possible sources of clinical heterogeneity are judged by combining the differences in population characteristics (e.g., comorbidity, age, gender, BMI, blood sugar indicators, DPN duration), intervention (e.g., different laser parameters such as energy density, wavelength, different intervention forms), outcome evaluation methods, and control selection in the studies included in the comparison of clinical knowledge, and then verified by subgroup analysis and sensitivity analysis.

#### Subgroup analysis

For the primary outcomes of NCV and clinical scores, the trials will be sub-grouped by dosage of LLLT such as energy density (greater than 3 J/cm^2^ or not), therapeutic schedules (whether or not sufficient treatment duration and sessions), BMI (≥ 24 kg/m^2^ or not), and blood sugar indicator of patients (FBG ≤ 7 mmol/L and 2hPBG ≤ 8 mmol/L, above this standard or no report)

#### Sensitivity analysis

In the sensitivity analysis, the following two types of studies will be excluded, one by one: (1) studies missing data, and (2) studies with a high risk-of-bias rating. The impacts of research quality, sample sizes, missing data, and statistical methods on the results of the meta-analysis will be evaluated by merging the data.

#### Reporting bias

If the number of studies is more than 10, a funnel plot will be constructed to examine publication bias.

#### Grading the quality of evidence

The quality of evidence for the entire study will be assessed using the Grading of Recommendations Assessment, Development and Evaluation (GRADE) approach [27]. The limitations of study design, inconsistency, indirect evidence, inaccuracy, and publication bias will be evaluated. The GRADE system classifies the evidence into four levels: high, moderate, low, or very low.

## Discussion

DM is a major cause of human mortality after malignant tumors and cardiovascular diseases. DPN is the most common chronic neurological complication of DM. The limited efficacy, adverse effects, and high costs of conventional pharmacological treatments have led both doctors and patients to seek effective and safe non-pharmacological treatments. Ever since Mester [28] proposed in 1966 that low-intensity laser can be used as a biological stimulus, researchers have been using lasers in clinical therapy. Many researchers have conducted clinical trials to assess the effectiveness of LLLT on improving symptoms and NCV in DPN, but there is currently no evidence-based review that confirms the efficacy and safety of LLLT for DPN. We will conduct this systematic review and meta-analysis to provide evidence that will allow doctors to better choose treatment methods for DPN. The primary outcomes that we have chosen are NCV as well as clinical scores that assess neurological function and related symptoms. Although diagnostic methods for DPN are diverse, NCV is a “benchmark” method for the assessment of DPN because of its repeatability and objectivity [29, 30]. Furthermore, various clinical scores are widely used to assess the severity of peripheral neuropathy.

Potential sources of heterogeneity in studies mainly include the LLLT parameters (e.g., energy density, wavelength, and continuous output power) and therapeutic schedules (e.g., treatment time, total sessions, and treatment sessions per week). Energy density is the most important factor related to the effects of LLLT. One study has suggested that LLLT at doses of 1.5 J/point and 3 J/point have no effect on pain in patients with knee osteoarthritis [31]. Another study reported that LLLT with a dose of 6 J/point was more effective than LLLT with a dose of 3 J/point for treating knee osteoarthritis [32]. If such heterogeneity is found in our assessment, a subgroup analysis will be conducted according to the applied LLLT energy density of the included studies. Wavelength is also considered to be an essential parameter for the effects of LLLT. A study has demonstrated that penetration is proportional to wavelength in the range of 450–1030 nm [33]. According to the World Association for Laser Therapy, wavelengths of 780–860 nm or 904 nm for LLLT are recommended for treating arthritis patients [34, 35]. We will therefore conduct a subgroup analysis based on whether the wavelength of the laser used in the included studies is within, below, or above this range. In addition to energy density and wavelength, differences in treatment plans may also affect heterogeneity. Daily treatment for 2 weeks or treatment every other day for 3–4 weeks is recommended [34, 35]. Thus, whether or not sufficient treatment duration and sessions were conducted in the included studies will also be a factor to consider for subgroup analysis.

An exhaustive literature search will be performed to identify studies aimed at assessing the effectiveness and safety of LLLT in treating DPN. One limitation of this review might be the restriction of the analysis to studies published in English or Chinese only. By integrating and analyzing the data from included studies, we hope to provide high-quality evidence of the efficacy and safety of LLLT for DPN, as a potential treatment option for doctors and patients.

## Data Availability

The data is available upon request from the corresponding author.

## Abbreviations

DPN: Diabetic peripheral neuropathy
DM: Diabetes mellitus
LLLT: Low-level laser therapy
RCTs: Randomized clinical trials
NCV: Nerve conduction velocity
QOL: Quality of life
SF-36: the Short Form36
EQ-5D: EuroQol five dimensions questionnaire
VAS: Visual analog scale
NPS: Neuropathic pain scale
NPQ: Neuropathic Pain Questionnaire
SF-MPQ: Short-form McGill Pain Questionnaire
BPI-DPN: Brief Pain Inventory for Diabetic Peripheral Neuropathy
FBG: Fasting blood glucose
2hPBG: 2 h postprandial blood glucose
HbA1c: Glycosylated hemoglobin
